# A characterization of four B16 murine melanoma cell sublines molecular fingerprint and proliferation behavior

**DOI:** 10.1186/1475-2867-13-75

**Published:** 2013-07-26

**Authors:** Corina Danciu, Alexandra Falamas, Cristina Dehelean, Codruta Soica, Heinfried Radeke, Lucian Barbu-Tudoran, Florina Bojin, Simona Cîntă Pînzaru, Melania F Munteanu

**Affiliations:** 1 Faculty of Pharmacy, University of Medicine and Pharmacy “Victor Babes”, EftimieMurgu Square, No. 2, 300041 Timişoara, România; 2 Biomedical Physics, Biomedical, Theoretical Physics, and Molecular Spectroscopy Department, Faculty of Physics, Babes-Bolyai University, Kogalniceanu 1, RO 400084 Cluj-Napoca, România; 3 Pharmazentrum Frankfurt/Center for Drug Research, Development and Safety, Clinic of J.W. Goethe University, Theodor-Stern-Kai 7, 60590 Frankfurt, Germany; 4 Electron Microscopy Center Faculty of Biology & Geology "Babes-Bolyai", University of Cluj-Napoca, 5-7 Clinicilor Street, 400006 Cluj-Napoca, Romania; 5 Department of Physiology and Immunology, University of Medicine and Pharmacy "Victor Babes", Eftimie Murgu Square, No. 2, 300041 Timisoara, Romania; 6 Department of Clinical Laboratory and Sanitary Chemistry, “Vasile Goldis” University, 1 Feleacului Str., Arad 310396 Romania

**Keywords:** B16 cells, SERS, TEM, MTT, Doubling time

## Abstract

**Background:**

One of the most popular and versatile model of murine melanoma is by inoculating B16 cells in the syngeneic C57BL6J mouse strain. A characterization of different B16 modified cell sub-lines will be of real practical interest. For this aim, modern analytical tools like surface enhanced Raman spectroscopy/scattering (SERS) and MTT were employed to characterize both chemical composition and proliferation behavior of the selected cells.

**Methods:**

High quality SERS signal was recorded from each of the four types of B16 cell sub-lines: B164A5, B16GMCSF, B16FLT3, B16F10, in order to observe the differences between a parent cell line (B164A5) and other derived B16 cell sub-lines. Cells were incubated with silver nanoparticles of 50–100 nm diameter and the nanoparticles uptake inside the cells cytoplasm was proved by transmission electron microscopy (TEM) investigations. In order to characterize proliferation, growth curves of the four B16 cell lines, using different cell numbers and FCS concentration were obtained employing the MTT proliferation assay. For correlations doubling time were calculated.

**Results:**

SERS bands allowed the identification inside the cells of the main bio-molecular components such as: proteins, nucleic acids, and lipids. An "on and off" SERS effect was constantly present, which may be explained in terms of the employed laser power, as well as the possible different orientations of the adsorbed species in the cells in respect to the Ag nanoparticles. MTT results showed that among the four tested cell sub-lines B16 F10 is the most proliferative and B164A5 has the lower growth capacity. Regarding B16FLT3 cells and B16GMCSF cells, they present proliferation ability in between with slight slower potency for B16GMCSF cells.

**Conclusion:**

Molecular fingerprint and proliferation behavior of four B16 melanoma cell sub-lines were elucidated by associating SERS investigations with MTT proliferation assay.

## Background

Skin cancer including both melanoma and the non-melanoma forms represent an increasing pathology among other types of cancer. Spectroscopic methods play a vital part in all areas of science and are the main tool of modern chemistry for the identification of molecular structures. They were applied in the last period for the diagnosis and pathology evolution surveillance on skin carcinoma, more frequent on non-melanoma types [1]. Raman spectroscopy is an analytical technique based on inelastic scattering of monochromatic light, usually from a laser source that allows chemical characterization of molecules in a sample. In recent years, the scientific world demanded a growing number of noninvasive diagnostic and nondestructive structure analysis tools; in consequence laser Raman spectroscopy has been playing an increasingly important role in analytical science, especially in biomedical analysis [2]. Each molecule has a unique fingerprint of Raman peaks [3,4], thus Raman spectroscopy has been successfully applied for the investigation of cells [5], biological tissues [6] as well as *in vivo* applications [7]. Raman signals can be enhanced by many orders of magnitude when the probed molecules are attached to metallic nanostructures (e.g. colloidal gold, silver nanoparticles) but with the observation that Raman scattering takes place in the high local optical fields of these structures [8]. Compared to the Raman signal of cells, the strongly amplified SERS signal allows much shorter acquisition times. In this manner, SERS can provide nano scale information about the biology of cells [9], enabling thus early diagnosis of diseases before the morphology changes. By introducing the silver nanoparticles inside the cells and acquiring SERS signal from the junctions between the metal nanoparticles and the adsorbed molecular components, information about the molecular composition of the investigated sample can be obtained. However, a complete understanding of the intracellular uptake, transport, metabolism and subcellular distribution of nanostructured materials remains limited. Nevertheless, overall findings reported that live cells trap nanoparticles of proper dimension in vesicles, hampering them to get into nucleus, although the SERS signals from cells were assigned mostly to vibrational modes of nucleus components. An “on and off” blinking effect has been reported for most experiments of this type [10], and the lack of reproducibility in SERS signal was rather judged as a need to optimize the nano architectures rather than taking into account the “live” system, i.e. live cell, where biological processes are rolling on.

Proliferation behavior has been analyzed by MTT (3-(4,5-dimethylthiazol-2-yl)-2,5-diphenyltetrazolium bromide) assay. It presents the advantage of being an inexpensive assay and among a variety of non-radioactive viability assessments, the MTT test developed by Mossmanis still one of the most versatile and popular assays [11]. In order to support this statement stand some of the latest articles in the field [12-15]. Regarding the cell lines involved in this study: B164A5 is a cell line derived from a skin melanoma of a C57BL/6 strain mouse, showing fibroblast-like characteristics, which produce melanin. Cells may lose ability to produce pigmentation in long term culture [16]. B16-F10 was derived from the parent B16 line by selection for the ability to form lung colonies *in vivo* after intra venous injection and subsequently established *in vitro* after 10 (B16-F10) cycles of lung colony formation [17]. B16-GMCSF cells represent a variant of the B16-F10 line transduced by using an MFG retroviral vector encoding murine GM-CSF [18]. It has been shown that vaccination with irradiated B16 tumor expressing granulocyte macrophage colony-stimulating factor (GM-CSF; Gvax) promotes rejection of established murine melanomas [19]. In case of B16-FLT 3, murine B16 melanoma cells where transfected with the gene for the *Flt3*-L cytokine (FLT3-Fvax), which also has the role of promoting the rejection of established murine melanomas [20].

We investigated here the nanoparticles uptake into live cells and the Raman amplification signal from the main bio molecular components present in four B16 murine cell lines, with the aim of correlating these findings with the cell growth and the chemical composition of each cell line. The study appraises the ability of the SERS technique to evaluate the cell status and chemical changes correlated with the spectroscopic signal. Such approach would be of crucial importance in early diagnosis of diseases and evaluation of therapy according to the morphological changes. In order to see their “behavior”, evaluation of cell growth curves was carried through MTT proliferation assay. Cell proliferation was assessed by monitoring the conversion of MTT to formazan, process catalyzed by mitochondrial dehydrogenase enzymes [11]. Previous studies on cancer cells have correlated results obtained by MTT assay with spectroscopic methods [21].

## Materials and methods

B164A5 cells were acquired from Sigma Aldrich (ECACC and Sigma Aldrich, origin Japan stored UK), B16F10, B16GMCSF, and B16FLT3 cell lines were kindly provided by Prof. Dr. Med. H. Radeke, Goethe Universitätsklinikum, Frankfurt am Main, Germany. Dulbecco’s Modified Eagle’s Medium (DMEM), Phosphate-buffered saline (PBS), Penicillin/Streptomycin mixture, HEPES 4-(2-hydroxyethyl)-1-piperazineethanesulfonic acid and Trypsin where acquired from Gibco; Fetal Calf Serum (FCS), EDTA, Trypan Blue, glutaraldeyde, AgNO_3,_ trisodium citrate where acquired from Sigma Aldrich; MTT cell proliferation kit was acquired from Roche, Germany. The complete growth medium for this cells is Dulbecco’s Modified Eagle’s Medium (DMEM), supplemented with 10% fetal calf serum (FCS), 1% Penicillin/Streptomycin mixture (Pen/Strep, 10,000 IU/ml) and 2% HEPES4-(2-hydroxyethyl)-1-piperazineethanesulfonic acid. The cells were cultured by incubation at 37°C in 5% CO_2_ atmosphere. When the confluence was 70-80% (every two or three days) the cells were passed using 0.25% Trypsin- 1 mM EDTA solution followed by centrifugation (5 minutes, 1200 rpm) and replated in T75 culture flasks at a sub-cultivation ratio of 1:10 to ensure optimal proliferation.

### Surface enhanced Raman scattering analysis

For the SERS analysis cells where washed with PBS, trypsinised, inactivated with medium Dulbecco’s Modified Eagle’s Medium (DMEM), supplemented with 10% fetal calf serum (FCS) and 1% Penicillin/Streptomycin mixture (Pen/Strep, 10,000 IU/ml) and 2% HEPES 4-(2-hydroxyethyl)-1-piperazineethanesulfonic acid, centrifuged 5 min. at 1200 rpm; medium was removed, cells where resuspended in new medium and replanted in Lab-Tek™ Chamber Slides, using a volume of 1 ml medium/ chamber and a number of 15.000 cells per each chamber. After 24 h cells where incubated with 50 μL colloidal silver in each chamber. After another 24 h of incubation medium was removed, cells where washed with PBS twice ant afterwards fixed with a 50–50 mixture (v/v) of methanol and acetone 10 minutes at -20C. The SERS measurements were performed on the culture chamber slides.

Silver colloid was prepared through the Lee Meisel method [22]. Briefly, 100 ml of a 1 mM AgNO_3_ three distilled aqueous solution was heated to 93-100°C and then 2 ml of a 1% trisodium citrate solution was added. The mixture was kept in constant (previously achieved) temperature for about 1 hour and then was allowed to cool down to the room temperature. The resultant colloidal mixture was of dark grey color. Finally, the colloidal solution was centrifuged at 1200 rotations/min, for 5 min.

For the SERS measurements a dispersive high performance micro-Raman spectrometer, BrukerSenterra with high spatial and spectral resolution was employed. The 633 nm laser line was focused on the cells through a 50x objective. The exposure time for each spectrum was 0.5 to 1 s and the spectra were acquired by focusing the laser at different points on the fixed cells. The power of the laser was set to 5 mW. Each spectrum was the result of 5 acquisitions.

### Transmission electron microscopy (TEM) analysis

In order to prove that nanoparticles of colloidal silver where incorporated into the cell TEM analyze was made. For the cells the same conditions where respected as for the SERS analysis: volume of 1 ml medium/ chamber and a number of 15.000 cells per each chamber. After 24 h cells from each chamber where incubated with 50 μL colloidal silver prepared after standard protocols. After another 24 h of incubation medium was removed, cells where washed with PBS twice. The cells attached to the membrane of Falcon Cell Culture Insert were prefixed for one hour with glutaraldeyde (2.5% in PBS), rinsed 3 times in PBS, and post fixed for one hour in osmium acid (2% in PBS). Dehydration was done in graded acetone in distilled water dilutions, followed by infiltration with Epon resin. Sections of about 100 nm, obtained on a diamond knife (Diatome) with Leica UC6 ultramicrotome were post-stained with lead citrate and uranyl acetate. The collodion-carbon coated 400 mesh Cu grids were examined with a Jeol JEM 1010 transmission electron microscope (JEOL, Japan).

### Doubling time

Briefly, for the determination of doubling time cells were counted with the haemocytometer using trypan blue. After trypsinization and centrifugation, 20 μl cell suspension was well mix with 20 μl trypan blue in an Eppendorf and from this mixture 20 μl were introduced in the counting camera of the haemocytometer. The number of live cells was counted using a hand tally counter. Afterwards it was calculated the total number corresponding to the suspension taken into study. The doubling time was measured for each cell line two times per week. The number of weeks taken into study was 4.

### MTT assay

For the spectrophotometric characterization of the four B16 cell lines by MTT 3-(4,5-Dimethylthiazol-2-yl)-2,5-diphenyltetrazolium bromide, a yellow tetrazole) assay 100 μl cell suspension containing: 15 000 cells, 10 000 cells, 6 000 cells, 3000 cells of the four types of B16 melanoma cells were seeded into a 96-well microplate and attached to the bottom of the well. After 24 hours 100 μL of new medium containing 10% FCS, 5% FCS, 1% FCS, and 0% FCS was added. Cells were kept in these conditions for 24 h. In day three 10 μl MTT reagent from a 5 mg/mL stock solution were introduced in each well. The intact mitochondrial reductase converted and precipitated MTT as purple crystals during a 4 h contact period. After four hours the precipitated crystals were dissolved in 100 μL of solubilisation solution. Finally, the reduced MTT was spectrophotometrically analyzed at 570 nm, using the 656 nm reference of an ELISA reader.

### Statistics

The Prism software package (Graph Pad Prism 4.03 for Windows) was used for data presentation. Data from at least three experiments are presented as mean ± SD. One way Anova followed by Bonferroni post-test was used to determine the statistical significance between various experimental groups; *, **, and *** indicate p<0.05, p<0.01 and p<0.001, respectively.

## Results and discussion

### TEM investigations

**Figure 1:**
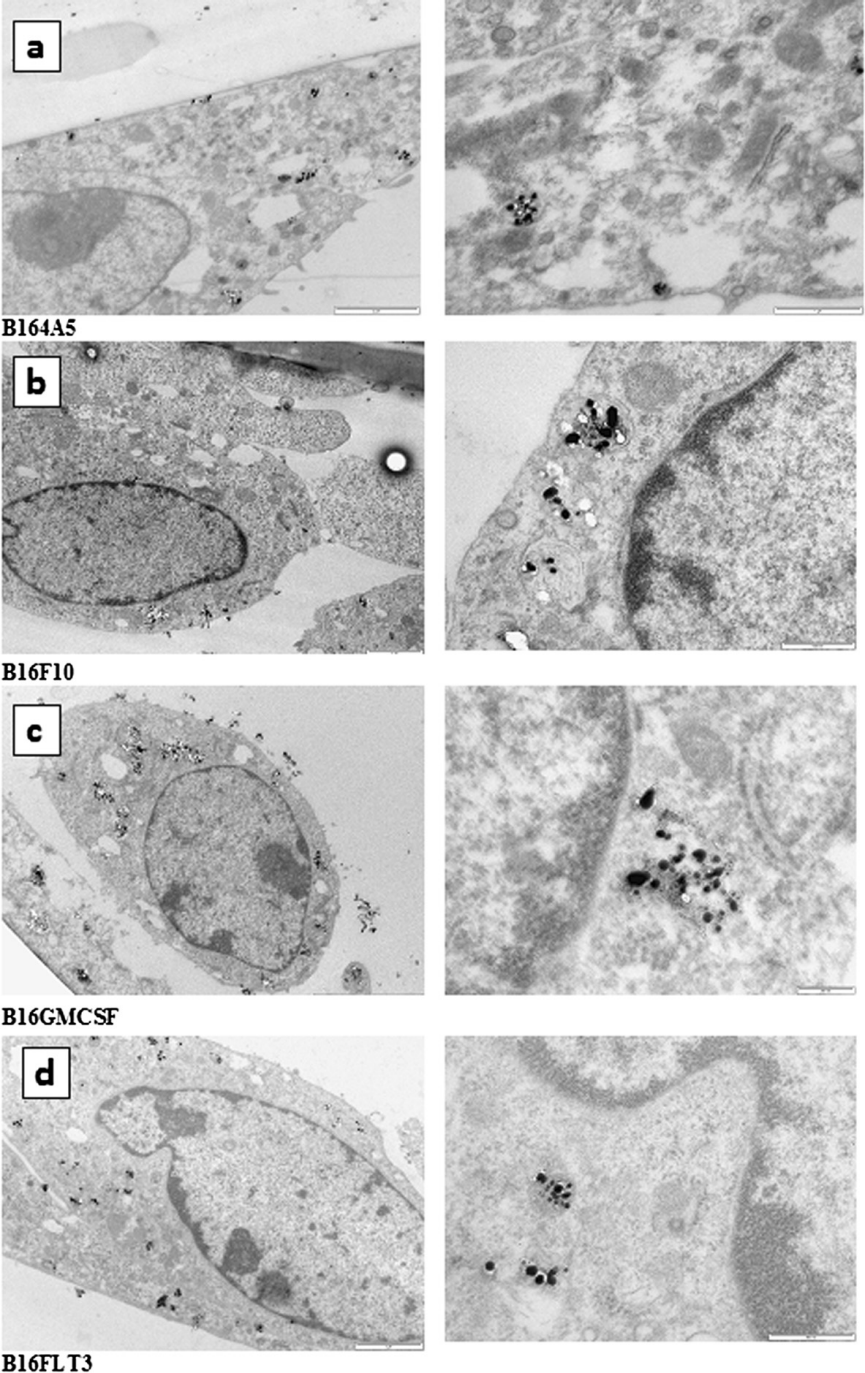
**TEM image of silver nanoparticles inside the cytoplasm. a** –B164A5 cells; **b** –B16F10 cells; **c** –B16GMCSF cells; **d **–B16FLT3 cells.

The colloidal nanoparticles present an absorption maximum at 420 nm and a full width at half maximum of 50 nm. TEM images of the silver colloid exhibited both spheroidal (50 to 100 nm diameter) and rod-like shapes with lengths ranging from 100 to 200 nm and about 30–40 nm rod diameter. The plasma membrane is a semi-permeable boundary between the cell and its outside environment, and the mechanisms employed for a particle to get into the cell are: diffusion, osmosis, active transport, and endocytosis. For small and non-polar molecules free diffusion is possible, instead bigger particles are incapable of crossing the plasma membrane and require uptake mechanisms such as endocytosis [23]. Due to negative charge of the cell surface, positively charged nanoparticles are preferentially taken up by living cells and the smaller particles were internalized by caveolin - independent pathway. The large nanoparticles penetrated the membrane by endocytosis, clathrin - dependent [24]. TEM observation revealed the nanoparticles uptake into the cytoplasm through both diffusion and endocytosis. Images demonstrate that the silver nanoparticles penetrated the cell membrane and were found inside the organelles and cytoplasm with perinuclear localization, free or surrounded by vesicles (Figure 1). Due to size of particles they cannot cross the nuclear pores and no particles were found in the cell nucleus. They mainly localized in the early and late lysosome, but they are also found in other organelles such as mitochondria and endoplasmic reticulum. Free aggregates appear by rapid uptake and lysosome by passing due to conjugating protein transduction domains to the surface of the nanoparticle. Localization at the level of mitochondria is realized by the presence of extra nuclear DNA [25]. These data are consistent with other experiments which underline that silver nanoparticles penetrate the cells membrane and can be used for the detection of the SERS signal [26,27]. Their aggregation is possible in the growth medium and due to their increased size endocytosis is assumed [28]. Analyzing a big number of cells from each of the four cell lines, it can be remarked that for all four B16 cell lines taken into study, incorporation was made both by diffusion and endocytosis, with no particular differences between the B16 cell lines. Pictures from each cell line can be observed in Figure 1 TEM images of silver nanoparticles inside the cytoplasm (Figure 1a –B164A5 cells; Figure 1b –B16F10 cells; Figure 1c –B16GMCSF cells; Figure 1d–B16FLT3 cells).

### SERS analysis

Prior to the acquisition of the SERS spectra, cells without silver colloid were also investigated. No signal was recorded from these cells, without any exception. When excited with the 785 nm laser line, the silver colloid incubated cells exhibited allow Raman signal, mostly covered by a broad fluorescence. Only an intense band located at 234 cm^-1^ band, which might indicate the silver nanoparticles attached to the intra-cellular components and a broad band centered at 1380 cm^-1^ (data not shown here). On the other hand, when the cells were excited with the 633 nm laser line, intense SERS spectra were acquired. This laser line falls closer to the absorption maximum of the colloidal nanoparticles, thus a better enhancement is observed.

**Figure 2:**
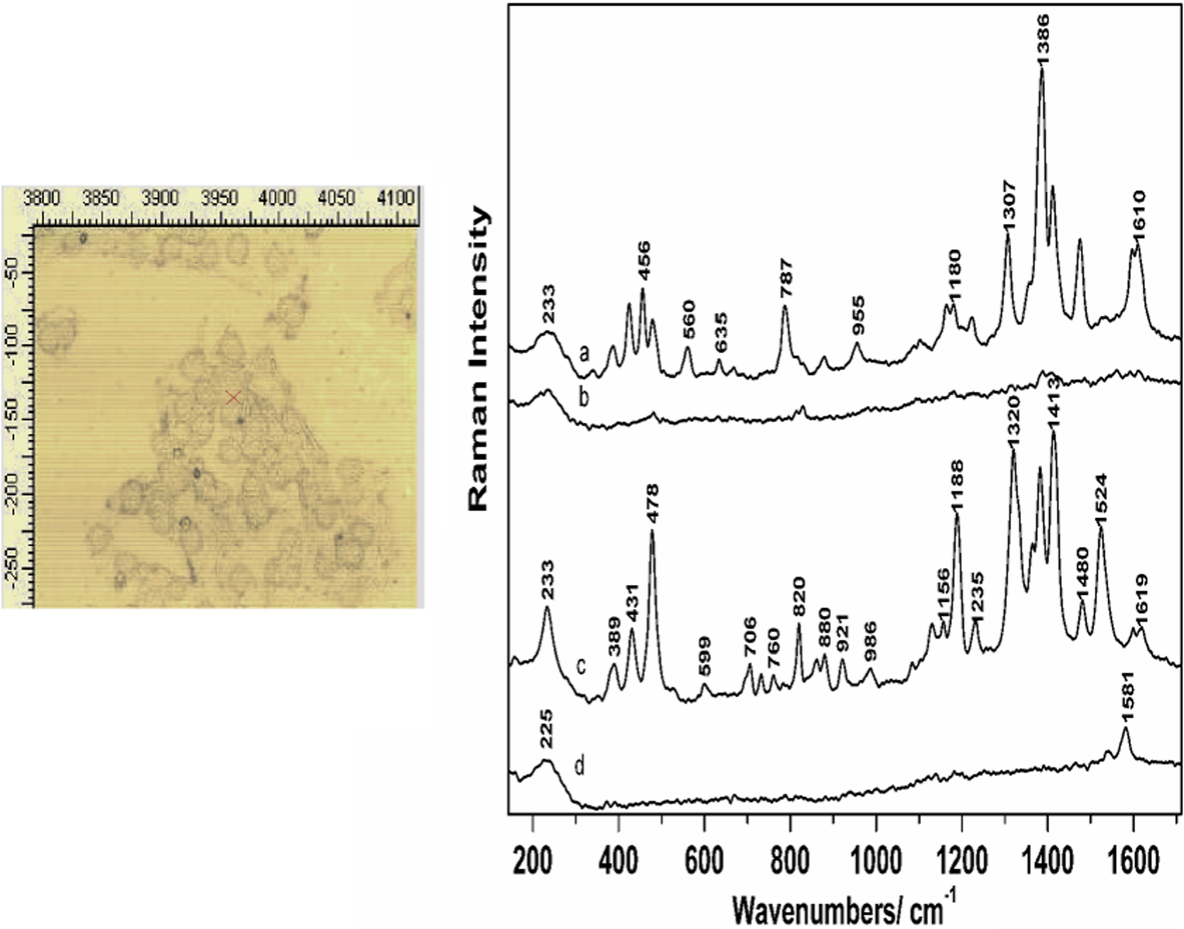
**Time dependence SERS spectra acquired from a B164A5 cell.** Excitation 633 nm. Laser power 5 mW.

The SERS signal collected from different points across a cell exhibited a specific spectral shape, consistent with the nanoparticles accumulation. An enhanced Raman signal was obtained from the molecular species in the close vicinity of the nanoparticles. Since the laser spot encompasses an area of signal collection which depends on the objective magnification (i.e. 50x, NA 0.45), both regions, with and without nanoparticles, can fall in the laser spot and scatter the radiation. However, an interesting phenomenon was observed even for the signal acquired from the same focal spot, upon multiple accumulations. Such changes are illustrated in Figure 2 for a B164A5 cell. (Figure 2 Time dependence SERS spectra acquired from a B164A5 cell. Excitation 633 nm. Laser power 5 mW) The SERS spectra show intense bursts of Stokes shifted photons in an “on and off” manner exhibiting considerable fluctuations in both signal intensities and frequencies, over the course of the acquisition of 4 spectra in a total of 40 s. This phenomenon known as "blinking SERS" has been observed by different research groups [29,30]. The sudden appearance and disappearance of the SERS spectra was evidenced mainly for single molecule SERS experiments [8].

Regardless of the fluctuations of the SERS signal from one acquisition to another, some bands could still be correlated. These are located at 233, 385, 431, 478, 1188, 1320, 1386, 1413, and 1620 cm^-1^. The amide I band of proteins around 1656 cm^-1^ is absent from the spectra, which is consistent with our previous observations regarding the SERS signals from biological molecules [31]. A tentative assignment of the main SERS bands is further proposed based on the current literature in the field [32]. The enhanced band at 227 cm^-1^ can be representative to the Ag-N mode when N-containing molecules are adsorbed to the Ag nanosurface. The 820 cm^-1^ band in spectrum c) could be assigned to out of plane vibrations of tyrosine. The 1188 cm^-1^ band in spectrum c), assigned to phenylalanine and in general to C-N stretching vibrations of proteins, is highly amplified, while in spectrum a) it has a much weaker intensity and appears shifted to 1180 cm^-1^. The 1232 cm^-1^ band may indicate the presence of the amide III band of proteins [33], while the 1620 cm^-1^ band seen in spectrum c) could be assigned to amide I vibrations, as well as C-C vibrations of tyrosine and tryptophan [34]. The 1320 cm^-1^ band observed in spectrum c), decreases in amplitude in spectrum a) and is shifted towards smaller wavenumbers. Similar situation is observed for the 1413 cm^-1^ band, which decreases in intensity in spectrum a) and the 1524 cm^-1^ band in spectrum c), which is completely absent from spectrum a).

Similar SERS bands were reported in the current literature based on SERS studies of cells. Kang *et al*. reported among others, the 1386, 1413, 1477, 1590, 1610 cm^-1^ bands and concluded while investigating cell apoptosis of HELA cells, that SERS signal strengths changed over time [35]. The 1386 cm^-1^ band was assigned to nucleotides, proteins, while the 1477 cm^-1^ band was attributed to CH_2_ and CH_3_ bending vibrations of lipids and proteins [10]. DNA contributions have also been observed in these spectra, such as the 787 cm^-1^ band, observed in spectrum a) and assigned to DNA backbone [33], as well as the 1413 cm^-1^ band assigned to amino acids and COO^-^ asymmetric stretching vibrations. From the analysis of the SERS spectra, we can conclude that the nanoparticles are found mostly in the vicinity or attached to proteins, as well as nucleic acids.

**Figure 3:**
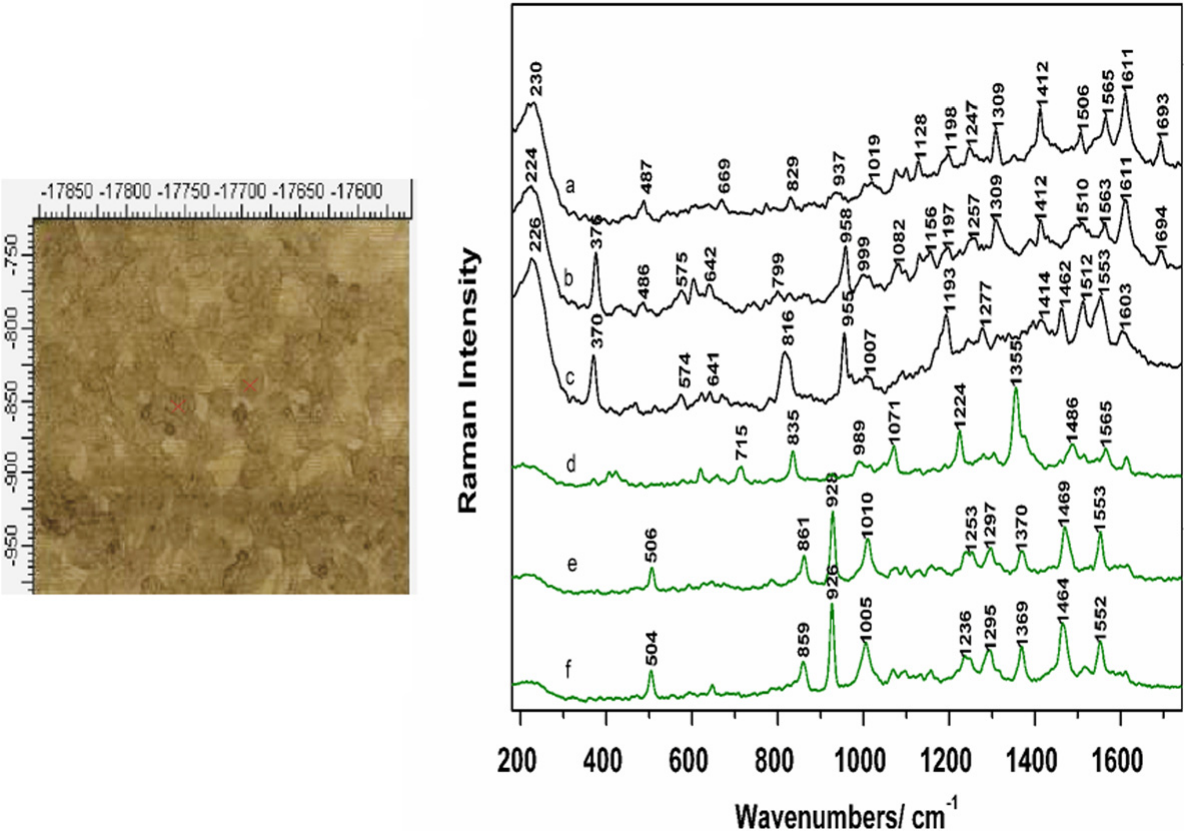
**Time dependence SERS spectra acquired from two different B16GMCSF cells.** Spectra **a)-c)** are representative for the first cell, while spectra **d)-f)** are characteristic for the second cell. Excitation 633 nm. Laser power 5 mW.

The nanoparticles were found in all other B16 cell lines investigated in this study and a tentative assignment of the main reproducible SERS bands indicated mostly the presence of proteins and nucleic acids. The SERS signal varied a lot from one acquisition to another, an effect which may be caused by the cellular events that take place in the live cells, as well by the inhomogeneity of the cellular composition and the nanoparticles distribution. Figure 3 Time dependence SERS spectra acquired from two different B16GMCSF cells. Spectra a)-c) are representative for the first cell, while spectra d)-f) are characteristic for the second cell. Figure 3 presents a time series dependence of SERS spectra acquired from the same spot focused on two different B16GMCSF cells. The spectra are presented in order to investigate the reproducibility of the signal from one acquisition to another and two cells were chosen to better observe the biochemical similarities and differences between cells of the same sub-line.

Some of the main bands seen in the spectra characteristic to the first cell (spectra a)-c) in Figure 3) are located at 224, 370, 574, 641, 955, 1193, 1309, 1414, 1506, 1563, 1611, and 1693 cm^-1^. However, slight wavenumber shifts and intensity fluctuations can be observed in these spectra from one acquisition to another. An intense band is observed around 224 cm^-1^ in all spectra. The 370 cm^-1^ band can be seen in the bottom two spectra as a sharp amplified band, while in the upper spectrum the bands disappears completely. The same case can be seen for the 574, 641, and 955 cm^-1^, while the latter vibrational mode appears shifted with 3 cm^-1^ in the middle spectrum. Some of the common bands in the SERS spectra are present at 1193, 1414, 1506, 1563, and 1611 cm^-1^. The 1193 cm^-1^ band can be assigned to nucleotides or proteins (tyrosine and phenylalanine), the 1414 cm^-1^ is assigned to amino acids, the 1506 cm^-1^ to nucleic acid bases, while the 1563 cm^-1^ is assigned to amide II band of proteins [10].

A similar situation is observed for the second cell, as well. Two bands appear in the bottom two spectra in the low wavenumber region, at 504 and 859 cm^-1^. The 859 cm^-1^ which is shifted in spectrum e) to 861 cm^-1^ is assigned to tyrosine/collagen. The same spectra show an intense 926 cm^-1^, which is completely absent in spectrum d). The phenylalanine band appears shifted from 1005 cm^-1^ in the bottom spectrum, to 1010 cm^-1^ in the middle one, and 989 cm^-1^ in spectrum d). Going towards higher wavenumbers, the same vibration modes can be seen in the bottom two spectra: 1236, 1253, 1295, 1369, 1464, and 1552 cm^-1^. Some of these, such as the 1355, 1486, and 1565 appear in spectrum d) as well, although slightly shifted and presenting fluctuations in their intensities. Based on the current literature in the field [33,34], a tentative assignment of these SERS modes, is proposed. Protein contributions can be observed at 1565 cm^-1^ assigned to amide II or tryptophan, at 1253 cm^-1^ characteristic to amide III, 1295 cm^-1^ assigned to CH deformation vibrations, 1071 cm^-1^ assigned to C-N stretching vibrations, the phenylalanine band at 1005 cm^-1^, and proline at 926 cm^-1^. Lipids contributions were observed at 1292 cm^-1^ characteristic of CH bending vibrations and nucleic acids were present at 1486 cm^-1^, assigned to guanine and adenine nucleic acid bases, and 832 cm^-1^ is assigned to DNA backbone vibrations.

**Figure 4:**
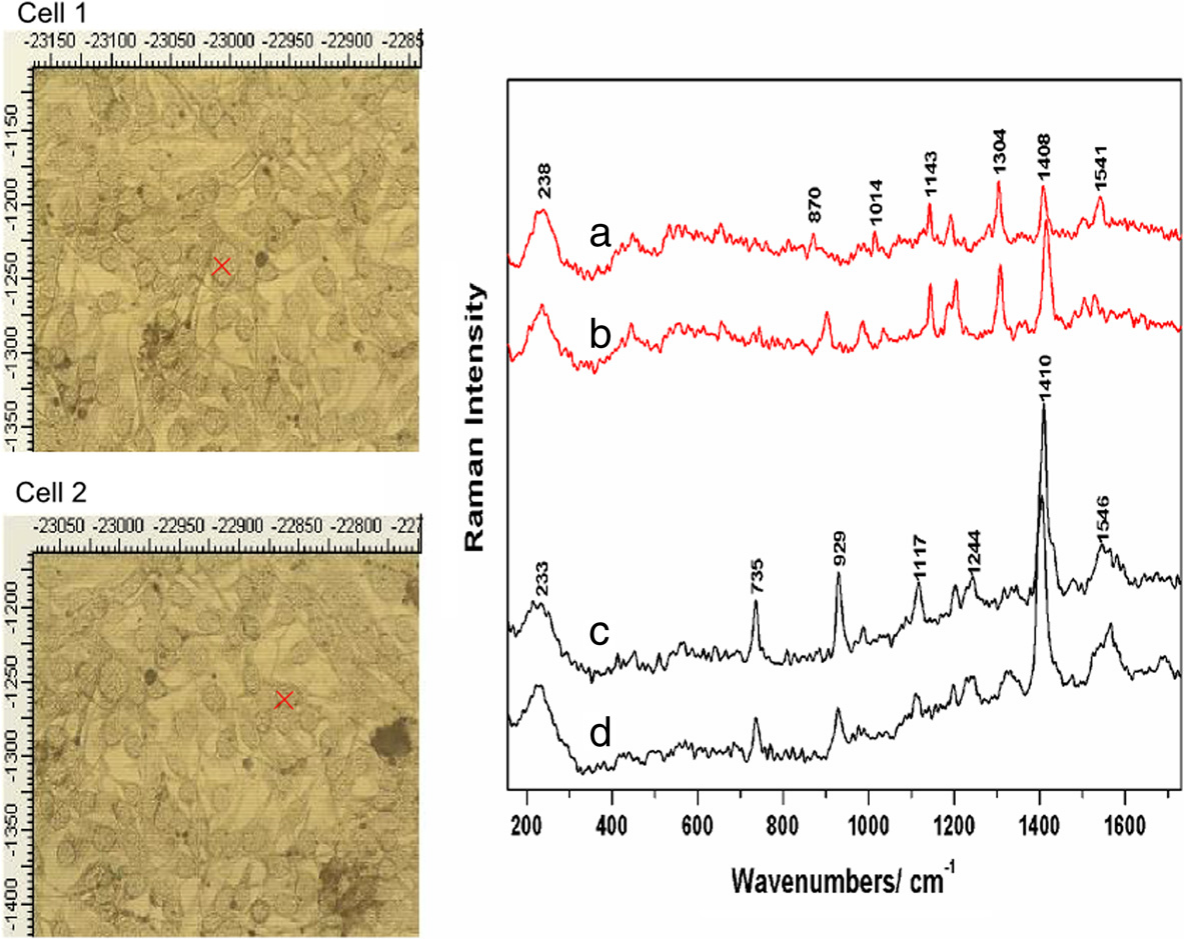
**Time dependence SERS spectra acquired from two different B16FLT3 cells.** Spectra **a)-b)** are representative for the first cell, while spectra **c)-d)** are characteristic to the second cell. Excitation 633 nm. Laser power 5 mW.

Figure 4 shows the SERS results characteristic to the B16FLT3 cell sub-line. The SERS spectra were recorded at 5 s interval from each, from two different cells. Figure 4 Time dependence SERS spectra acquired from two different B16FLT3 cells. Spectra a)-b) are representative for the first cell, while spectra c)-d) are characteristic to the second cell. The SERS spectra acquired from the first investigated cell present apart from the 238 cm^-1^, the 443, 1144, 1204, 1308, and 1415 cm^-1^ common bands. Although, slight wavenumber shifting can still be observed, as is the case of 1308 or 1415 cm^-1^, the wavenumber shifts are not as big as in previous cases. The second investigated cell presented even more reproducible SERS spectra. Some of the common bands seen here are located at 233, 735, 929, 1117, 1244, 1410, and 1546 cm^-1^. These were reported by other research groups working with cells and nanoparticles and they were assigned to proline at 929 cm^-1^, C-N stretching vibrations of proteins at 1144 cm^-1^, tryptophan or phenylalanine at 1204 cm^-1^, amide III or adenine and thymine nucleic acids at 1241 cm^-1^, deoxyribose COO^-^ stretching vibrations at 1415 cm^-1^, and tryptophan at 1546 cm^-1^[32-34]. The spectral differences observed in the 1700–1500 cm^-1^ range, where apart from the amide I band, C-C and C=N stretching modes are present, can be assigned to specific biochemical changes due to cell proliferation [31].

**Figure 5:**
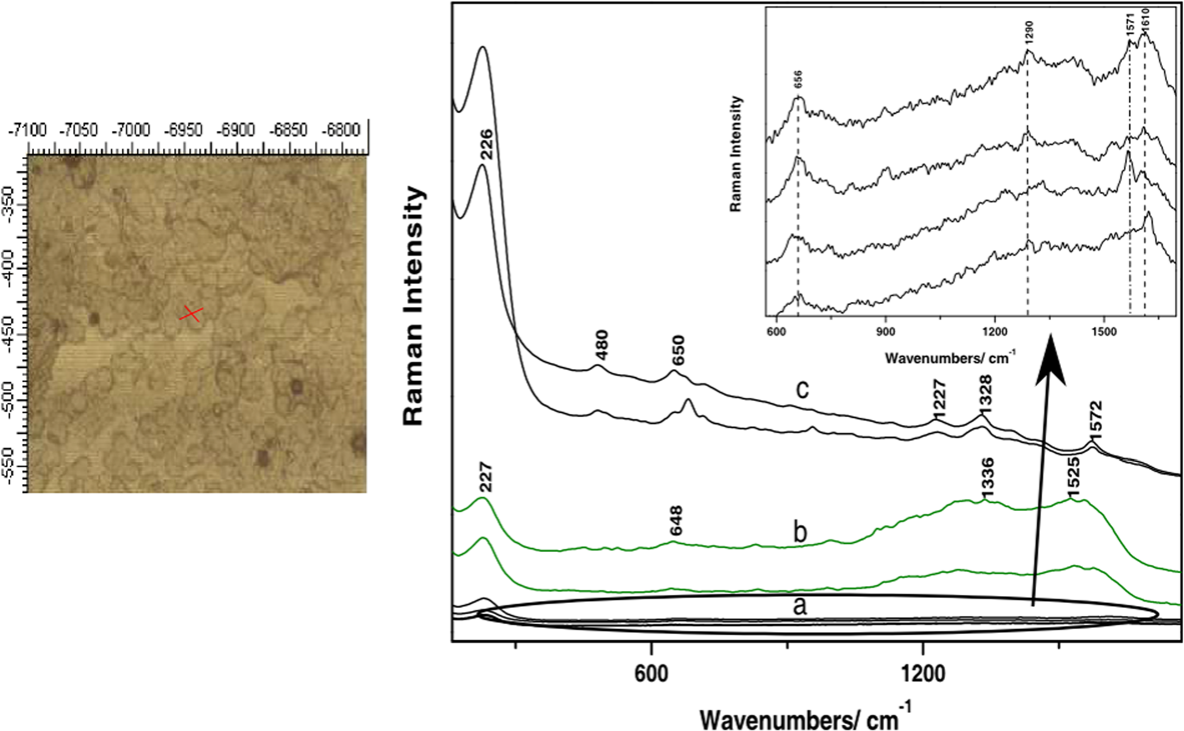
**Typical SERS spectra collected from the B16F10 cells upon incubation with Ag nanoparticles.** Group **a)** and **b)** represents signals collected from two distinct cells, whereas **c)**, **d)** and **e)** are spectra collected from melanoma skin tissue (B16 cells injection) incubated with nanoparticles. The spectral details of a) are shown in the right side. Excitation 633 nm. Laser power 5 mW

The last analyzed cell line was B16F10. These cells presented particularly weak SERS signal. The spectra presented in Figure 5 show typical SERS signal acquired from this line. Figure 5 Typical SERS spectra collected from the B16F10 cells upon incubation with Ag nanoparticles. Group a) and b) represent signals collected from two distinct cells, whereas c-e are spectra collected from melanoma skin tissue (B16 cells injection) incubated with nanoparticles. For the investigation of this cell sub-line, the spectra were acquired by moving the laser spot from one place to another in a conglomerate of cells. The recorded signal was different compared to the previous cell lines and presented more background noise than usual. The SERS spectra collected from the two groups of cells presented here show similar vibrational modes, regardless of the fact that the spectra collected from the second group of cells, are very weak in intensity. The strongest band observed in these spectra is located at 230 cm^-1^. Other bands are observed at 648, 995, and the two broad bands in the fingerprint region centered at 1299, 1336, and 1525 and 1555 cm^-1^, respectively. Some of these bands were reported by previous SERS studies of cells, as well [10,34] and were assigned to amide II, tryptophan (1555 cm^-1^), CH_2_, CH_3_ bending vibrations of proteins (1336 cm^-1^), proteins amide III (995 cm^-1^), CCH bending and ring breathing vibration modes of tyrosine or O-P-O stretching vibration of DNA/RNA (830 cm^-1^). Based on our previous studies focusing on the investigation of the SERS signal from tissues [36], we believe that the clearly resolved bands around 1300 cm^-1^ are attributable to the nucleic species.

Looking at the overall shape of these spectra, we noticed they resemble well the SERS spectra acquired from *ex vivo* mice skin tissue samples immersed in colloidal silver solution (group c) in Figure 5). The autopsy tissues samples were collected from a mice specimen injected with B16 cells. The tissue characteristic reproducible SERS bands are located at 1573, 1330, 1231, 955, 681, 481, and 236 cm^-1^[37]. The spectra acquired from the cells are presented here in comparison to tissue SERS spectra, in order to point out the similarity between them and thus, the bio-molecular composition between these melanin producing cells and the skin tissue.

### MTT proliferation assay

After characterizing the four B16 cell sub-lines from a spectroscopic point of view growth curves of the four B16 cell lines where analyzed employing the MTT proliferation assay. For a better characterization of the proliferative activity also doubling time was calculated using the formula presented below, where *q*_1_ represents the number of cells at time *t*_1_ and *q*_2_ represents the number of cellsat time *t*_2_.

$$T_{d}=\left(t_{2}-t_{1}\right)*\frac{\log\left(2\right)}{\log\left(\frac{q2}{q1}\right)}.$$

**Figure 6:**
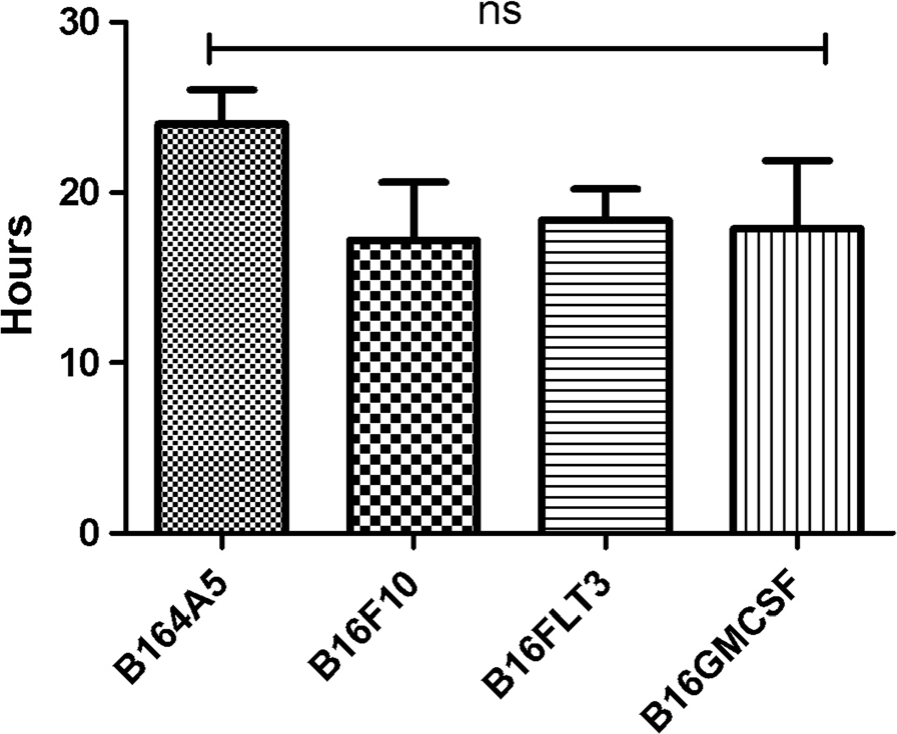
Doubling time of the four B16 cell sublines as revealed by trypan blue assay.

Assuming a constant growth rate (increase per unit of time is proportional to the current quantity), the results showed that B164A5 cells present the longest doubling time, 24 h. The lowest value vas obtained for B16F10 cells, 17.2 h. For the transduced B16GMCSF cells doubling time was 17.9 h and for the transfected B16FLT3 cells the value was 18.4 h. The first conclusion after this assay is that B16F10 cells have the smallest doubling time so the biggest multiplication ratio while B164A5 cells have the biggest doubling time so the smallest multiplication ratio. B16GMCSF cells and B16FLT3 cells present values in between, with a slight higher value in the case of B16FLT3 cells. Results can be seen in Figure 6 Values obtained for the doubling time of the four cell lines. In another study Ohira *et al*. found the doubling time of B16F10 cells 20.1 h, while Yerlikaya *et al*. found the doubling time of B16F10 cells 14.2 h [38,39]. Probably variable values are found by different research groups because of different chosen compositions of the growing medium. Qarawi *et al*. estimated that the doubling time for B16 cells is approximately 24 h [40].

**Figure 7:**
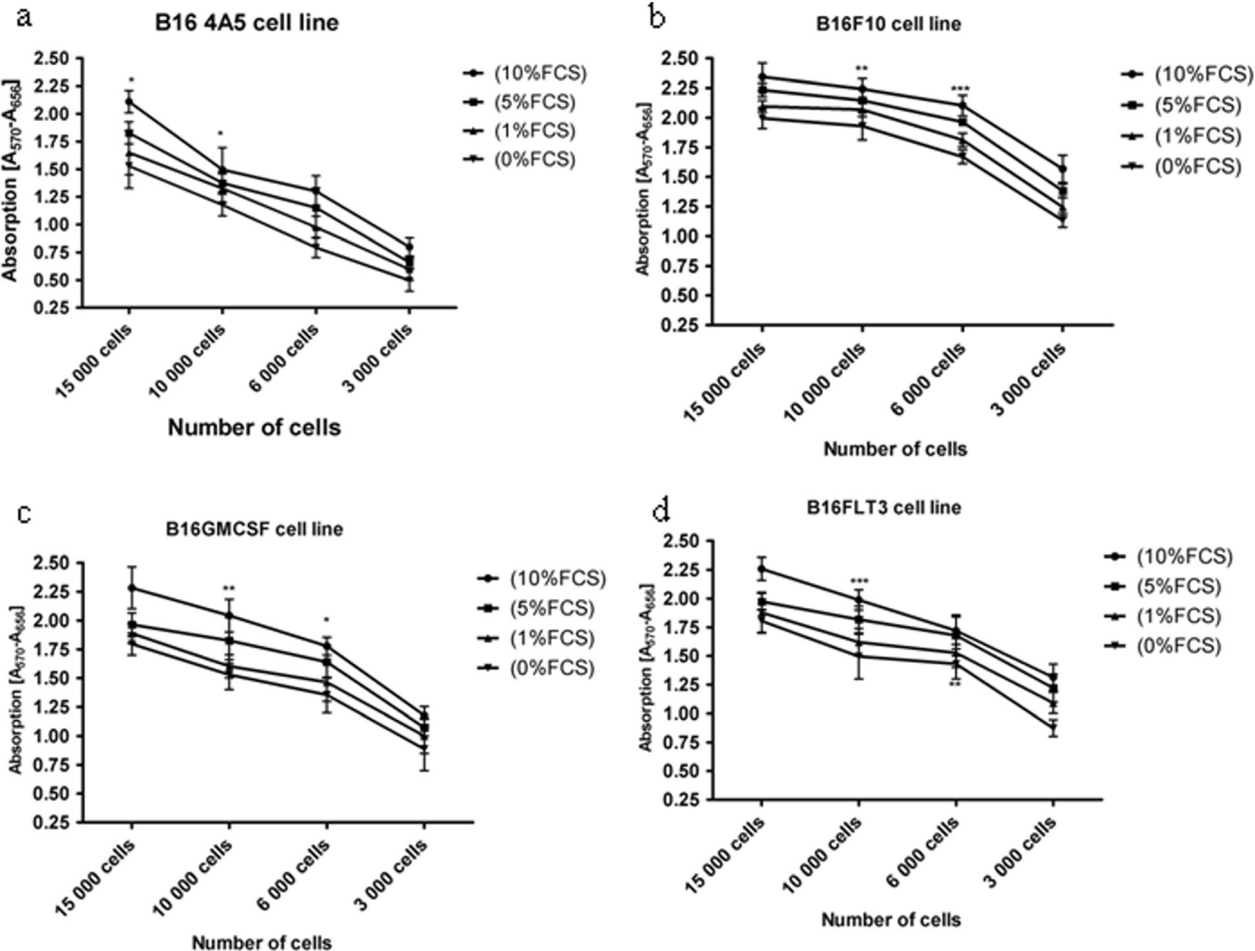
(a,b,c,d): Growth curves employing different number of cells and different FCS concentrations corresponding to a) B164A5 cell line; b) B16F10 cell line; c) B16GMCSF cell line; d) B16FLT3 cell line.

After doubling time was found for each cell line, MTT assay was employed in order to characterize the growth curves. Different number of cells 15 000 cells, 10 000 cells, 6000 cells, 3000 cells of each cell line and also different fetal calf serum (FCS) concentrations: 10%, 5%, 1%, and 0% where used as variable parameters. FCS is the most widely used growth supplement for cell culture media of eukaryotic cells because of its high content of embryonic growth promoting factors and low level of antibodies. Changing the optimal concentration of FCS provided in protocols is directly proportional with changes in cell proliferation [41,42]. Results can be seen in Figure 7a, b, c, d: Absorption using different concentrations of FCS a) Of four different numbers of B164A5 cells b) Of four different numbers of B16F10 cells c) Of four different numbers of B16GMCSF cells d) Of four different numbers of B16FLT3 cells

MTT results show a linear relationship between the number of cells and the absorbance with a higher absorbance corresponding to the biggest number of cells taken into study. MTT proliferation assay delivered curves with the typical four-phase course for different cell numbers: lag period (immediately after reseeding), exponential growth -log phase, during which the cell population doubles over a definable period, (doubling time-characteristic for each cell line), plateau or stationary phase, where the growth fraction drops to close to zero, the death phase [43].

Beside the cells number, FCS concentration was also analyzed. Cells were cultured without FCS or with diminished supplementation of FCS (i.e., 1%, 5%) in comparison to normal culture conditions (i.e., 10% FCS). A decrease value of the absorbance was directly proportional to a decreased fetal calf serum concentration in the medium. These findings are valid for all four cell lines, with slight differences regarding the values for the absorbance (using the same number of cells) depending on the cell line. Starving cells prior to treatment was found to be a method to sensitize cells to treatment and result in increased cytotoxicity of active agents [44-46]. Other groups used serum starvation in order to synchronize cells to the same cell cycle stage [47-49].

One can observe a direct correlation between the doubling time and the values of the absorbance, corresponding to each cell line, in the same experimental conditions (number of cells/FCS concentration): higher values of the absorbance where found in case of B16F10, cells that have the lowest doubling time, so the highest rate of proliferation. On the opposite, lower values of the absorbance can be noticed in B164A5, cells that have the highest doubling time, so the lowest rate of proliferation. B16FLT 3 cells and B16GMCSF cells present values in between, with slight slower values for B16GMCSF. Taking in consideration data from the literature that characterize these four cell lines (e.g. how they were obtained), results are relevant: B16 F10 is a high metastatic cell line [17,50] and the data from this study show that in the same conditions (number of cells/FCS concentration) among the tested cell lines B16F10 presents the highest values for the absorbance. B16-GM cells, a variant of the B16-F10 cells transduced by using an MFG retroviral vector encoding murine GM-CSF [18,51] present, the second highest value for the absorbance. These values are followed by the ones obtained from B16FLT3 cells. In case of B16-FLT 3, murine B16 melanoma cells where transfected with the gene for the *Flt3*-L cytokine (FLT3-Fvax), which also has the role of promoting the rejection of established murine melanomas [20]. The lower values for the absorbance where noticed in case of B164A5 cell line -derived from a melanoma from the skin of a C57BL/6 strain mouse, which produces melanin [52,53]. For all four cell lines the percentage of death cells in the mentioned conditions was less than 3%.

## Conclusions

The SERS experiments presented here were conducted as an attempt to characterize the molecular components of four B16 murine melanoma cell sub-lines. These investigations provide a certain insight into the SERS behavior of these cells, demonstrating that information regarding mainly the proteins and nucleic acids in the cells can be obtained when inoculating them with silver nanoparticles. The observed SERS bands are different from one cell line to another, revealing different NPs uptake ability. Also, each particular cell exhibits specific SERS signal. Expanding to collective samples, the SERS investigation could surprise each cell in a specific evolution moment of its lifetime, associated with different cell cycle phases, hence the intense blinking effect. The blinking effect could also appear due to the in homogeneity of the cellular components. These results could be a useful tool for the investigation of the action of different anti-proliferative/cytotoxic agents. However, further experiments are needed in order to elucidate the complex composition of these cells and the differences between the modified B16 cell sub-lines from a spectroscopic point of view.

Specific growth curves for each cell sub-line were detected employing the MTT proliferation assay using both different cell numbers and fetal calf serum concentration as variable parameters. Doubling time for each cell line was also calculated, and results were in agreement with the information obtained from MTT. This data allowed us to conclude that the B16F10 is the most proliferative cell line and B164A5 has the lower growth capacity. Regarding B16FLT3 cells and B16GMCSF cells, they present proliferation ability in between with slight slower values for B16GMCSF. This proliferation capacity of the four B16 cell lines is in agreement with the way the cell sub-lines where obtained. MTT data can be correlated with the surface enhanced Raman investigations for a detailed characterization of different cell lines.

## Competing interests

The authors declare that they have no competing interests.

## Authors’ contributions

CD(T) and CS had contributions to conception and design of the study, acquisition of MTT data , analysis and interpretation of these data, AF and SCP had contributions to conception and design of the study, acquisition of SERS data, analysis and interpretation of these data, LBT and FB had contributions for preparation of the cells and for TEM analyse, CD, HR and MM had contributions for drafting the article and revising it critically for important intellectual content. All authors read and approved the final manuscript.
